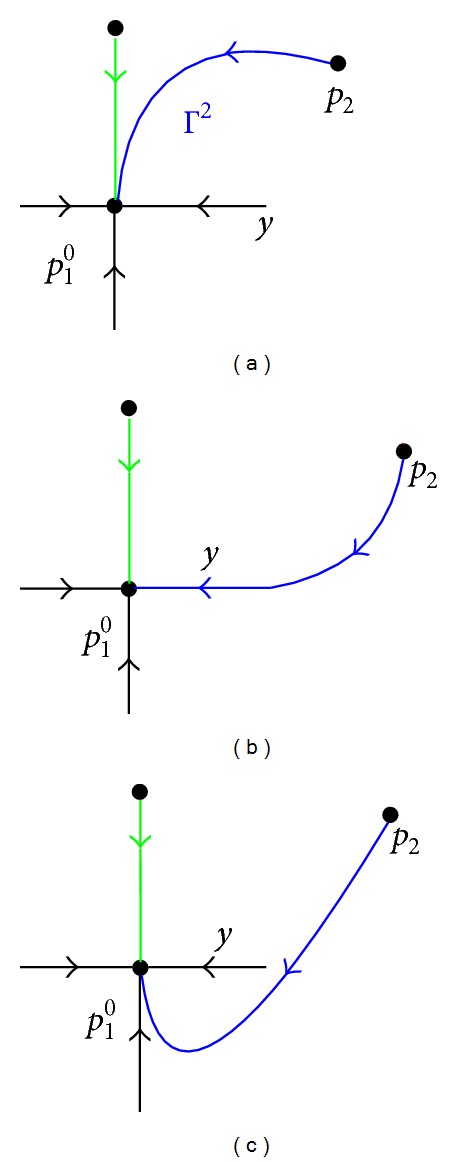# Bifurcations of Orbit and Inclination Flips Heteroclinic Loop with Nonhyperbolic Equilibria

**DOI:** 10.1155/2014/585609

**Published:** 2014-03-23

**Authors:** Fengjie Geng, Junfang Zhao

**Affiliations:** School of Science, China University of Geosciences (Beijing), Beijing, 100083, China

## Abstract

The bifurcations of heteroclinic loop with one nonhyperbolic equilibrium and one hyperbolic saddle are considered, where the nonhyperbolic equilibrium is supposed to undergo a transcritical bifurcation; moreover, the heteroclinic loop has an orbit flip and an inclination flip. When the nonhyperbolic equilibrium does not undergo a transcritical bifurcation, we establish the coexistence and noncoexistence of the periodic orbits and homoclinic orbits. While the nonhyperbolic equilibrium undergoes the transcritical bifurcation, we obtain the noncoexistence of the periodic orbits and homoclinic orbits and the existence of two or three heteroclinic orbits.

## 1. Introduction

In recent years, a great deal of mathematical efforts has been devoted to the bifurcation problems of homoclinic and heteroclinic orbits with high codimension, for example, the bifurcations of homoclinic or heteroclinic loop with orbit flip, the bifurcations of homoclinic or heteroclinic loop with inclination flip, and so forth; see [1–5] and the references therein. However, most of these papers considered the bifurcation problems of orbits connecting hyperbolic equilibria, and limited work has been done in the corresponding problems with nonhyperbolic equilibria; see [6–8]. To fill this gap, we investigate the bifurcations of orbit and inclination flip heteroclinic orbits with one nonhyperbolic equilibrium and one hyperbolic saddle. The method is using the fundamental solution matrix of the linear variational system to obtain the Poincaré  map, which is easier to get the bifurcation equations.

Consider the following *C* ^*r*^ (*r* ≥ 5) system

(1) $$\begin{array}{c} \dot{z}=g\left(z,\lambda ,\mu \right) \end{array}$$

and its unperturbed system

(2) $$\begin{array}{c} \dot{z}=f\left(z\right), \end{array}$$

where *z* ∈ ℝ^4^, the vector field  *g*  depends on the parameters (*λ*, *μ*), *λ* ∈ ℝ, *μ* ∈ ℝ^*l*^, *l* ≥ 2, 0 ≤ *λ*, |*μ* | ≪1, *g*(*z*, 0,0) = *f*(*z*), *g*(*p*_1_, 0, *μ*) = 0, and *g*(*p*_2_, *λ*, *μ*) = 0. Moreover, the parameter  *λ*  governs bifurcation of the nonhyperbolic equilibrium, while  *μ*  controls bifurcations of the heteroclinic orbits.

Assuming system (2) has a heteroclinic loop Γ connecting its two equilibria *p*_1_, *p*_2_, where Γ = Γ^1^⋃Γ^2^, Γ^*i*^ = {*z* = *r*_*i*_(*t*) : *t* ∈ ℝ}, *r*_*i*_(+*∞*) = *r*_*i*+1_(−*∞*) = *p*_*i*+1_, *i* = 1,2, *r*_3_(*t*) = *r*_1_(*t*), and *p*_3_ = *p*_1_. Furthermore, the linearization  *Df*(*p*_1_)  has real eigenvalues  0, *λ*_1_^1^, −*ρ*_1_^1^, and − *ρ*_1_^2^  satisfying − *ρ*_1_^2^ < −*ρ*_1_^1^ < 0 < *λ*_1_^1^; *Df*(*p*_2_) has simple real eigenvalues *λ*_2_^1^, *λ*_2_^2^, −*ρ*_2_^1^, and −*ρ*_2_^2^ fulfilling −*ρ*_2_^2^ < −*ρ*_2_^1^ < 0 < *λ*_2_^1^ < *λ*_2_^2^.

The following conditions hold in the whole paper:

(*H*_1_) (3) $$\begin{array}{c} e_{i}^{\pm }=\underset{t\to \pm \infty }{\lim}\frac{{\dot{r}}_{i}\left(-t\right)}{\left|{\dot{r}}_{i}\left(-t\right)\right|}, \end{array}$$

where *e*_1_^+^ ∈ *T*_*p*_1__ *W*_1_^*cu*^, *e*_1_^−^ ∈ *T*_*p*_2__ *W*_2_^*ss*^, *e*_2_^+^ ∈ *T*_*p*_2__ *W*_2_^*u*^, *e*_2_^−^ ∈ *T*_*p*_1__ *W*_1_^*s*^, and *e*_1_^−^ ∈ *T*_*p*_2__ *W*_2_^*ss*^ mean that Γ^1^ is a heteroclinic orbit with orbit flip, *W*_1_^*cu*^ is the center unstable manifold of *p*_1_, *W*_*i*_^*u*^ (resp., *W*_*i*_^*s*^) is the unstable (resp., stable) manifold of *p*_*i*_, and *W*_*i*_^*uu*^ (resp., *W*_*i*_^*ss*^) is the strong unstable (resp., stable) manifold of *p*_*i*_, *i* = 1,2. Moreover,

(4) dim⁡(Tr1(t)W1c∩Tr1(t)W2s)  =dim⁡(Tr1(t)W1cu∩Tr1(t)W2s)=1.

(*H*_2_) (5) $$\begin{array}{c} \underset{t\to +\infty }{\lim}T_{r_{1}(t)}W_{1}^{cu}=\mathrm{span}\left\{e_{1}^{-},T_{p_{2}}W_{2}^{uu}\right\},\\ \underset{t\to +\infty }{\lim}T_{r_{2}(t)}W_{2}^{u}=\mathrm{span}\left\{e_{2}^{-},T_{p_{1}}W_{1}^{u}\right\},\\ \underset{t\to -\infty }{\lim}T_{r_{1}(t)}W_{2}^{s}=\mathrm{span}\left\{e_{1}^{+},T_{p_{1}}W_{1}^{ss}\right\},\\ \underset{t\to -\infty }{\lim}T_{r_{2}(t)}W_{1}^{s}=\mathrm{span}\left\{e_{2}^{+},T_{p_{2}}W_{2}^{s}\right\}, \end{array}$$

where the first three equations mean that the center unstable manifold *W*_1_^*cu*^ of *p*_1_, the stable (resp., unstable) manifold *W*_2_^*s*^ (resp., *W*_2_^*u*^) of  *p*_2_  are fulfilling the strong inclination property. And the fourth equation implies that the stable manifold *W*_1_^*s*^ is of inclination flip as *t* → −*∞*.

It is worthy of noting that, for any integers *m* ≥ 1 and *n* ≥ 1, if we assume dim⁡(*W*_1_^*u*^) = dim⁡(*W*_2_^*uu*^) = *m* and dim⁡(*W*_1_^*ss*^) = dim⁡(*W*_2_^*ss*^) = *n*, then all the results achieved in this paper are still valid.

Let *λ* ∈ ℝ be a parameter to control the transcritical bifurcation of system (1), let the *x*-axis be the tangent space of the center manifold at *p*_1_, and let *θ*(*x*, *λ*, *μ*) be the vector field defined on the center manifold; then by [9], we may assume

(*H*_3_) *θ*(*x*_*p*_1__, *λ*, *μ*) = 0, (∂*θ*/∂*x*)(*x*_*p*_1__, 0,0) = 0, (∂^2^ *θ*/∂*x* ^2^)(*x*_*p*_1__, 0,0) > 0, (∂^2^ *θ*/∂*x*∂*λ*)(*x*_*p*_1__, 0,0) < 0, (∂^2^ *θ*/∂*x*∂*μ*)(*x*_*p*_1__, 0, *μ*) = 0, where *x*_*p*_1__ is the *x* component of *p*_1_.

If (*H*_3_) is true, then system (1) exhibits the transcritical bifurcation, that is, when *λ* > 0 (or *λ* < 0; in this paper, we only consider the case *λ* > 0; for the case *λ* < 0, one may discuss it similarly); there are two hyperbolic saddles *p*_1_^0^ and *p*_1_^1^ bifurcated from *p*_1_. Denote by *p*_1_^0^ = *p*_1_ = (0,0, 0,0)* and *p*_1_^1^ = *p*_1_ + (*λ*_*p*_, 0,0, 0)*, where *λ*_*p*_ = *θ*_0_ *λ* + *O*(*λ* ^2^) + *O*(*λμ*) and *θ*_0_ = −(∂^2^ *θ*/∂*x*∂*λ*)(*x*_*p*_1__, 0,0)/(∂^2^ *θ*/∂*x* ^2^)(*x*_*p*_1__, 0,0). Moreover, dim⁡(*W*_*p*_1_^0^_^*s*^) = 3, dim⁡(*W*_*p*_1_^0^_^*u*^) = 1, and dim⁡(*W*_*p*_1_^1^_^*u*^) = dim⁡(*W*_*p*_1_^1^_^*s*^) = 2.

The present paper is built up as follows. In Section 2, we devote it to deriving the successor functions by constructing a suitable Poincaré Map. The analysis to the bifurcations of system (2) is presented in Section 3, where we establish the existence of the heteroclinic loop, the homoclinic orbits, and the three or two heteroclinic orbits and the coexistence of a periodic orbit and a homoclinic loop, and the difference between the heteroclinic loop with hyperbolic equilibria and nonhyperbolic equilibria is revealed.

## 2. Normal Form and Poincaré Map

Let the neighborhood *U*_*i*_ of *p*_*i*_ be small enough and straight the local manifolds of *W*_*i*_^*s*^, *W*_2_^*uu*^, *W*_*i*_^*ss*^, and *i* = 1,2 in the neighborhood *U*_*i*_. And then by virtue of the invariance of these manifolds and a scale transformation *x* → *θ*_*xx*_^−1^(*x*_*p*_1__, 0,0)*x* and *λ* → −*θ*_*xλ*_^−1^(*x*_*p*_1__, 0,0)*λ*, system (1) has the following expression in  *U*_1_:

(6) x˙=−λpx+x2+O(u)[O(y)+O(v)]+O(x)[O(y)+O(u)+O(v)]+O(x)O(x2),y˙=[−ρ11(α)+⋯]y+O(v)[O(x)+O(u)],u˙=[λ11(α)+⋯]u+O(x)[O(y)+O(v)],v˙=[−ρ12(α)+⋯]v+O(y)[O(x)+O(y)+O(u)],

and in *U*_2_ it takes the following form:

(7) $$\begin{array}{c} \dot{x}=\left[\lambda_{2}^{1}\left(\alpha \right)+\cdots \right]x+O\left(u\right)\left[O\left(y\right)+O\left(v\right)\right],\\ \dot{y}=\left[-\rho_{2}^{1}\left(\alpha \right)+\cdots \right]y+O\left(v\right)\left[O\left(x\right)+O\left(u\right)\right],\\ \dot{u}=\left[\lambda_{2}^{2}\left(\alpha \right)+\cdots \right]u+O\left(x\right)\left[O\left(x\right)+O\left(y\right)+O\left(v\right)\right],\\ \dot{v}=\left[-\rho_{2}^{2}\left(\alpha \right)+\cdots \right]v+O\left(y\right)\left[O\left(x\right)+O\left(y\right)+O\left(u\right)\right], \end{array}$$

where *α* = (*λ*, *μ*), *λ*_*p*_ = *λ* + *O*(*λ* ^2^) + *O*(*λμ*), *λ*_1_^1^(0) = *λ*_1_^1^, *ρ*_*i*_^*j*^(0) = *ρ*_*i*_^*j*^, *j* = 1,2,  *i* = 1,2, *λ*_2_^*j*^(0) = *λ*_2_^*j*^,  *j* = 1,2.

From the normal form (6), (7), and the condition (*H*_1_), we may select −*T*_*i*_ and *T*_*i*_ such that

(8) $$\begin{array}{c} r_{1}\left(-T_{1}\right)={\left(\delta ,0,0,0\right)}^{*},\;\;r_{1}\left(T_{1}\right)={\left(0,0,0,\delta \right)}^{*},\\ r_{1}\left(-T_{2}\right)={\left(\delta ,0,0,\delta_{u},0\right)}^{*},\;\;r_{2}\left(T_{2}\right)={\left(0,\delta ,0,\delta_{v}\right)}^{*}, \end{array}$$

where *δ* > 0 is small enough such that {(*x*, *y*, *u*, *v*):|*x* | , |*y* | , |*u* | , |*v* | <2*δ*} ⊂ *U*_*i*_ and |*δ*_*u*_| = *o*(*δ*), |*δ*_*v*_ | = *o*(*δ*).

Consider the linear variational system

(9)_𝑖_ $$\begin{array}{c} \dot{z}=Df\left(r_{i}\left(t\right)\right)z \end{array}$$

and its adjoint system

(10)_𝑖_ $$\begin{array}{c} \dot{\phi }=-{\left(Df\left(r_{i}\left(t\right)\right)\right)}^{*}\phi , \end{array}$$

*i* = 1,2, where (*Df*(*r*_*i*_(*t*)))* is the transposed matrix of *Df*(*r*_*i*_(*t*)).

Supposing *Z*_*i*_(*t*) = (*z*_*i*_^1^(*t*), *z*_*i*_^2^(*t*), *z*_*i*_^3^(*t*), *z*_*i*_^4^(*t*)) is a fundamental solution matrix of  ((9)_*i*_), then, we arrive at the following lemma.

**Lemma 1**

If conditions (*H*_1_)–(*H*_3_) are satisfied, then

(1) there exists a fundamental solution matrix of (9)_1_ satisfying

(11) z11(t)∈(Tr1(t)W1cu)c∩(Tr1(t)W2s)c,z12(t)=−r˙1(t)|r˙1(T1)|∈Tr1(t)W1c∩Tr1(t)W2s,z13(t)∈Tr1(t)W1cu∩(Tr1(t)W2s)c,z14(t)∈(Tr1(t)W1cu)c∩Tr1(t)W2s

such that

(12) $$\begin{array}{c} Z_{1}\left(-T_{1}\right)=\left(\begin{array}{cccc} w_{1}^{11} & w_{1}^{21} & 0 & w_{1}^{41}\\ w_{1}^{12} & 0 & 0 & w_{1}^{42}\\ w_{1}^{13} & w_{1}^{23} & 1 & w_{1}^{43}\\ 0 & 0 & 0 & w_{1}^{44} \end{array}\right),\;\\ Z_{1}\left(T_{1}\right)=\left(\begin{array}{cccc} 1 & 0 & w_{1}^{31} & 0\\ {\tilde{w}}_{1}^{12} & 0 & w_{1}^{32} & 1\\ 0 & 0 & w_{1}^{33} & 0\\ 0 & 1 & w_{1}^{34} & 0 \end{array}\right); \end{array}$$

(2)  (9)_2_ has a fundamental solution matrix fulfilling

(13) z21(t)∈(Tr2(t)W2u)c∩(Tr2(t)W1s)c,z22(t)=−r˙2(t)|r˙2(T2)|∈Tr2(t)W2u∩Tr2(t)W1s,z23(t)∈Tr2(t)W2u∩(Tr2(t)W1s)c,z24(t)∈(Tr2(t)W2u)c∩Tr2(t)W1s

such that

(14) $$\begin{array}{c} Z_{2}\left(-T_{2}\right)=\left(\begin{array}{cccc} w_{2}^{11} & w_{2}^{21} & 0 & w_{2}^{41}\\ 0 & 0 & 0 & w_{2}^{42}\\ w_{2}^{13} & w_{2}^{23} & 1 & w_{2}^{43}\\ w_{2}^{14} & 0 & 0 & 0 \end{array}\right),\\ Z_{2}\left(T_{2}\right)=\left(\begin{array}{cccc} 1 & 0 & w_{2}^{31} & 0\\ 0 & 1 & w_{2}^{32} & 0\\ 0 & 0 & w_{2}^{33} & 0\\ {\tilde{w}}_{2}^{14} & w_{2}^{24} & w_{2}^{34} & 1 \end{array}\right), \end{array}$$

where *w*_*i*_^21^ < 0, *w*_1_^12^ *w*_*i*_^33^ *w*_2_^14^ *w*_2_^42^ ≠ 0, |(*w*_*i*_^33^)^−1^ *w*_*i*_^3*j*^ | ≪1, *j* = 1,2, 4, *i* = 1,2.

Now, let (*z*_*i*_^1^(*t*), *z*_*i*_^2^(*t*), *z*_*i*_^3^(*t*), *z*_*i*_^4^(*t*)) be a new local active coordinate system along Γ^*i*^. Given Φ_*i*_(*t*) = (*ϕ*_*i*_^1^(*t*), *ϕ*_*i*_^2^(*t*), *ϕ*_*i*_^3^(*t*), *ϕ*_*i*_^4^(*t*)) = (*Z*_*i*_^−1^(*t*))*, then Φ_*i*_(*t*) is the fundamental solution matrix of ((10)_*i*_), *i* = 1,2.

Let *z* = *r*_*i*_(*t*) + *Z*_*i*_(*t*)*N*_*i*_(*t*)≜*h*_*i*_(*t*), where *N*_*i*_(*t*) = (*n*_*i*_^1^, 0, *n*_*i*_^3^, *n*_*i*_^4^)*, *i* = 1,2. Defining the cross sections

(15) Si0={z=hi(−Ti):|x|,|y|,|u|,|v|<2δ},Si1={z=hi(Ti):|x|,|y|,|u|,|v|<2δ}

of  Γ_*i*_ at *t* = −*T*_*i*_  and *t* = *T*_*i*_, respectively, *i* = 1,2.

Now that if *q*_*i*_^0^ ∈ *S*_*i*_^0^ and *q*_*i*_^1^ ∈ *S*_*i*_^1^, then

(16) $$\begin{array}{c} q_{i}^{0}={\left(x_{i}^{0},y_{i}^{0},u_{i}^{0},v_{i}^{0}\right)}^{*}=r_{i}\left(-T_{i}\right)+Z_{1}\left(-T_{i}\right)N_{i}\left(-T_{i}\right),\\ N_{i}\left(-T_{i}\right)={\left(n_{i}^{0,1},0,n_{i}^{0,3},n_{i}^{0,4}\right)}^{*},\\ q_{i}^{1}={\left(x_{i}^{1},y_{i}^{1},u_{i}^{1},v_{i}^{1}\right)}^{*}=r_{i}\left(T_{i}\right)+Z_{i}\left(T_{i}\right)N_{i}\left(T_{i}\right),\\ N_{i}\left(T_{i}\right)={\left(n_{i}^{1,1},0,n_{i}^{1,3},n_{i}^{1,4}\right)}^{*}. \end{array}$$

Based on the expressions of *Z*_*i*_(−*T*_*i*_) and *Z*_*i*_(*T*_*i*_), we get their new coordinates of *q*_*i*_^0^(*n*_*i*_^0,1^, 0, *n*_*i*_^0,3^, *n*_*i*_^0,4^)* and *q*_*i*_^1^(*n*_*i*_^1,1^, 0, *n*_*i*_^1,3^, *n*_*i*_^1,4^)*; that is,

(17) n10,1=(w112)−1[y10−w142(w144)−1v10],n10,3=u10−w113(w112)−1y10+[w113w142(w112)−1−w143](w144)−1v10,n10,4=(w144)−1v10,x10=δ+w111n10,1+w141n10,4≈δ,n11,1=x11−w131(w133)−1u11,n11,3=(w133)−1u11,n11,4=y11−w~112x11+(w~112w131−w132)(w133)−1u11,v11≈δ,n20,1=(w214)−1v20,n20,3=u20−δ2u−w213(w214)−1v20−w243(w242)−1y20,n20,4=(w242)−1y20,x20≈δ,n21,1=x01−w231(w233)−1u01,n21,3=(w233)−1u01,n21,4=v01−δ2v−w~214x01+(w~214w231−w234)(w233)−1u01,y01≈δ.

Next, we divide our establishment of the Poincaré  map in the new coordinate system in three steps.

First, consider the map *F*_*i*_^1^ : *S*_*i*_^0^ ↦ *S*_*i*_^1^. Put *z* = *h*_*i*_(*t*) into (1); we have

(18) r˙i(t)+Z˙i(t)Ni(t)+Zi(t)N˙i(t) =g(ri(t)+Zi(t)Ni(t),λ,μ) =g(ri(t),0,0)+gz(ri(t),0,0)Zi(t)Ni(t)  +gλ(ri(t),0,0)λ+gμ(ri(t),0,0)μ+h.o.t. =f(ri(t))+Df(ri(t))Zi(t)Ni(t)  +gλ(ri(t),0,0)λ+gμ(ri(t),0,0)μ+h.o.t.

According to the fact ${\dot{r}}_{i}(t)=f(r_{i}(t))$ and ${\dot{Z}}_{i}(t)=Df(r_{i}(t))Z_{i}(t)$, it then yields to that

(19) N˙i(t)=Zi−1(t)[gλ(ri(t),0,0)λ+gμ(ri(t),0,0)μ]+h.o.t.

Integrating the above equation from −*T*_*i*_ to *T*_*i*_, we arrive at

(20) Ni(Ti)=Ni(−Ti)+∫−TiTiZi−1(t)gλ(ri(t),0,0)λ dt+∫−TiTiZi−1(t)gμ(ri(t),0,0)μ dt+h.o.t.

Noticing that Φ_i_*(*t*) = *Z*_*i*_^−1^(*t*), then

(21)_𝑖_ $$\begin{array}{c} n_{i}^{1,j}=n_{i}^{0,j}+M_{i\lambda }^{j}\lambda +M_{i\mu }^{j}\mu +\text{h}.\text{o}.\text{t}.,\;j=1,3,4, \end{array}$$

where

(22)_𝑖_ $$\begin{array}{c} M_{i\lambda }^{j}=\overset{T_{i}}{\underset{-T_{i}}{\int }}\phi_{i}^{j*}g_{\lambda }\left(r_{i}\left(t\right),0,0\right)dt,\\ M_{i\mu }^{j}=\overset{T_{i}}{\underset{-T_{i}}{\int }}\phi_{i}^{j*}g_{\mu }\left(r_{i}\left(t\right),0,0\right)dt,\;j=1,3,4. \end{array}$$

Together with (17) and ((21)_*i*_), ((22)_*i*_) then defines the map *F*_*i*_^1^ : *S*_*i*_^0^ ↦ *S*_*i*_^1^, (*n*_*i*_^0,1^, 0, *n*_*i*_^0,3^, *n*_*i*_^0,4^)↦(*n*_*i*_^1,1^, 0, *n*_*i*_^1,3^, *n*_*i*_^1,4^).

Next, to construct the map *F*_*i*_^0^ : *S*_*i*−1_^1^ ↦ *S*_*i*_^0^ (where  *S*_0_^1^ = *S*_2_^1^). Let *τ*_*i*_,  *i* = 1,2 be the flying time from *q*_*i*−1_^1^(*x*_*i*−1_^1^, *y*_*i*−1_^1^, *u*_*i*−1_^1^, *v*_*i*−1_^1^)* to *q*_*i*_^0^(*x*_*i*_^0^, *y*_*i*_^0^, *u*_*i*_^0^, *v*_*i*_^0^)*; set *s*_1_ = *e* ^−*ρ*_1_^1^(*α*)*τ*_1_^ and *s*_2_ = *e* ^−*ρ*_2_^1^(*α*)*τ*_2_^. By virtue of the approximate solution of system (6) and (7), if we neglect the higher terms, then the expression of *F*_1_^0^ : *S*_0_^1^ ↦ *S*_1_^0^ is

(23) $$\begin{array}{c} x_{0}^{1}\approx \frac{x_{1}^{0}}{h\left(s_{1}\right)},\;\;y_{1}^{0}\approx s_{1}y_{0}^{1},\\ \;\;\;u_{0}^{1}\approx s_{1}^{\lambda_{1}^{1}(\alpha )/\rho_{1}^{1}(\alpha )}u_{1}^{0},\;\;v_{1}^{0}\approx s_{1}^{\rho_{1}^{2}(\alpha )/\rho_{1}^{1}(\alpha )}v_{0}^{1} \end{array}$$

and *F*_2_^0^ : *S*_1_^1^ ↦ *S*_2_^1^ is

(24) $$\begin{array}{c} x_{1}^{1}\approx s_{2}^{\lambda_{2}^{1}(\alpha )/\rho_{2}^{1}(\alpha )}x_{2}^{0},\;\;y_{2}^{0}\approx s_{2}y_{1}^{1},\\ u_{1}^{1}\approx s_{2}^{\lambda_{2}^{2}(\alpha )/\rho_{2}^{1}(\alpha )}u_{2}^{0},\;\;v_{2}^{0}\approx s_{2}^{\rho_{2}^{2}(\alpha )/\rho_{2}^{1}(\alpha )}v_{1}^{1}, \end{array}$$

where (*s*_*i*_, *u*_*i*_^0^, *v*_*i*−1_^1^), *i* = 1,2 are called Shilnikov coordinates, and

(25) $$\begin{array}{c} h\left(s\right)=\{\begin{array}{cc} {\left(\lambda_{p}\right)}^{-1}\left[x_{1}^{0}-\left(x_{1}^{0}-\lambda_{p}\right)s^{\lambda_{p}/\rho_{1}^{1}(\alpha )}\right], & \lambda_{p}\neq 0,\\ 1-{\left(\rho_{1}^{1}\left(\alpha \right)\right)}^{-1}x_{1}^{0}\ln s, & \lambda_{p}=0. \end{array} \end{array}$$

**Figure 1:**
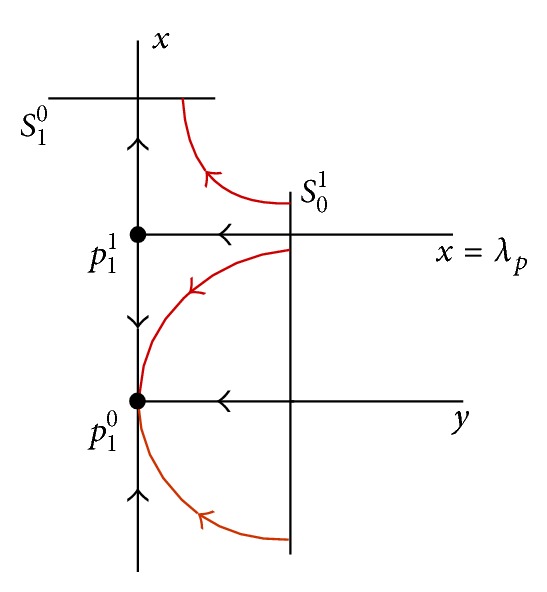


Since the nonhyperbolic equilibrium *p*_1_ undergoes a transcritical bifurcation based on the structure of orbits in *U*_1_, we may see that the equation *x*_0_^1^ ≈ *x*_1_^0^/*h*(*s*_1_) holds only for *x*_0_^1^ ≥ *λ*_*p*_. While for *x*_0_^1^ ∈ [−*β*, *λ*_*p*_)  (0 < *β* ≪ 1), the map *F*_1_^0^ is well defined only if *s*_1_ = 0 (see Figure 1). So, we extend the domain of *F*_1_^0^, defining

(26) $$\begin{array}{c} x_{1}^{0}=\delta ,\;s_{1}=0,\;\text{if}\;x_{0}^{1}\in \left[-\beta ,\lambda_{p}\right). \end{array}$$

The final step is to compose the maps *F*_*i*_^0^ and *F*_*i*_^1^, and then *F*_1_ = *F*_1_^1^∘*F*_1_^0^ : *S*_0_^1^ ↦ *S*_1_^1^ can be expressed as

(27) n11,1=(w112)−1δs1−(w112)−1w142(w144)−1s1ρ12(α)/ρ11(α)v01+M1λ1λ+M1μ1μ+h.o.t.,n11,3=u10−w113(w112)−1δs1+[w113w142(w112)−1−w143](w144)−1s1ρ12(α)/ρ11(α)v01+M1λ3λ+M1μ3μ+h.o.t.,n11,4=(w144)−1s1ρ12(α)/ρ11(α)v01+M1λ4λ+M1μ4μ+h.o.t.

and *F*_2_ = *F*_2_^1^∘*F*_2_^0^ : *S*_1_^1^ ↦ *S*_2_^1^( = *S*_0_^1^) as

(28) n21,1=(w214)−1δs2ρ22(α)/ρ21(α)+M2λ1λ+M2μ1μ+h.o.t.,n21,3=u20−δ2u−w213(w214)−1δs2ρ22(α)/ρ21(α)−w243(w242)−1s2y11+M2λ3λ+M2μ3μ+h.o.t.,n21,4=(w244)−1s2y11+M2λ4λ+M2μ4μ+h.o.t.

Set *G*_*i*_ = *F*_*i*_(*q*_*i*−1_^1^) − *q*_*i*_^1^, *i* = 1,2. Combing ((21)_*i*_), (23), (24), (27), and (28), we derive the successor functions *G*_*i*_^*j*^:

(29) G11=(w112)−1δs1−δs2λ21(α)/ρ21(α)+M1λ1λ+M1μ1μ+h.o.t.,G13=u10−w113(w112)−1δs1−(w133)−1s2λ22(α)/ρ21(α)u20+M1λ3λ+M1μ3μ+h.o.t.,G14=(w144)−1s1ρ12(α)/ρ11(α)v01−y11+w~112δs2λ21(α)/ρ21(α)+M1λ4λ+M1μ4μ+h.o.t.,G21=(w214)−1δs2ρ22(α)/ρ21(α)−δh(s1)+w231(w233)−1s1λ11(α)/ρ11(α)u10+M2λ1λ+M2μ1μ+h.o.t.,G23=u20−δ2u−w213(w114)−1δs2ρ22(α)/ρ21(α)−w243(w242)−1s2ρ21(α)/ρ21(α)y11−(w233)−1s1λ11(α)/ρ11(α)u10+M2λ3λ+M2μ3μ+h.o.t.,G24=(w244)−1s2y11−v01+δ2v+w~214δh(s1)−(w234−w~214w231)(w233)−1s1λ11(α)/ρ11(α)u10+M2λ4λ+M2μ4μ+h.o.t.

It is easy to see that what we need to do is considering the solutions of

(30) $$\begin{array}{c} \left(G_{1}^{1},G_{1}^{3},G_{1}^{4},G_{2}^{1},G_{2}^{3},G_{2}^{4}\right)=0 \end{array}$$

with *s*_1_ ≥ 0 and *s*_2_ ≥ 0. This is because the solution of (30) with *s*_1_ = *s*_2_ = 0 (resp., *s*_1_ > 0, *s*_2_ > 0; *s*_1_ = 0, *s*_2_ > 0 or *s*_1_ > 0, *s*_2_ = 0) means that system (1) has a heteroclinic loop (resp., a periodic orbit; homoclinic loop).

## 3. Main Results

Based on the expressions of the successor functions and the implicit function theorem, we know that the equation (*G*_1_^3^, *G*_1_^4^, *G*_2_^3^, *G*_2_^4^) = 0 has a unique solution (*u*_1_^0^, *u*_2_^0^, *y*_1_^1^, *v*_0_^1^). And putting it into (*G*_1_^1^, *G*_2_^1^) = 0, then we obtain the following bifurcation equations:

(31) (w112)−1δs1−δs2λ21/ρ21+M1λ1λ+M1μ1μ+h.o.t.=0,(w214)−1δs2ρ22/ρ21−δh(s1)+M2λ1λ+M2μ1μ+w231(w233)−1s1λ11/ρ11[w113(w112)−1δs1+(w133)−1         ×s2λ22/ρ21(δ2u−M2λ3λ−M2μ3μ)         −M1λ3λ−M1μ3μ]+h.o.t.=0.

Firstly, we consider the case *λ* = 0, which means the transcritical bifurcation does not happen. By (23) and (25), (31) turns to

(32) (w112)−1δs1−δs2λ21/ρ21+M1μ1μ+h.o.t.=0,(w212)−1δs2ρ22/ρ21−δ1−(ρ11)−1δln⁡s1+M2μ1μ+w231(w233)−1w113(w112)−1δs1λ11/ρ11+1−w231(w233)−1s1λ11/ρ11M1μ3μ+h.o.t.=0.

Noticing that *λ*_1_^1^/*ρ*_1_^1^ > 0, which shows lim⁡_*s*_1_→0_ *s*_1_^*λ*_1_^1^/*ρ*_1_^1^^(1 − (*ρ*_1_^1^)^−1^ *δ*ln⁡*s*_1_) = 0, it then follows that

(33) $$\begin{array}{c} s_{1}-w_{1}^{12}s_{2}^{\lambda_{2}^{1}/\rho_{2}^{1}}+\delta^{-1}w_{1}^{12}M_{1\mu }^{1}\mu +\text{h}.\text{o}.\text{t}.=0,\\ {\left(w_{2}^{12}\right)}^{-1}s_{2}^{\rho_{2}^{2}/\rho_{2}^{1}}-\frac{1}{1-{\left(\rho_{1}^{1}\right)}^{-1}\delta \ln s_{1}}+\delta^{-1}M_{2\mu }^{1}\mu +\text{h}.\text{o}.\text{t}.=0. \end{array}$$

From the above bifurcation equations, we obtain the following results immediately.

**Theorem 2**

Let the conditions (*H*_1_)–(*H*_3_) be true and *M*_*iμ*_^1^ ≠ 0, *i* = 1,2. Then, for *λ* = 0 and 0 < |*μ*| ≪ 1, one has

(i) for rank⁡(*M*_1*μ*_^1^, *M*_2*μ*_^1^) = 2, there exists a codimension 2 surface

(34) $$\begin{array}{c} L_{12}=\left\{\mu :M_{1\mu }^{1}\mu +h.o.t.=M_{2\mu }^{1}\mu +h.o.t.=0\right\} \end{array}$$

such that system (1) has a unique heteroclinic loop near Γ if and only if *μ* ∈ *L*_12_, where the surface *L*_12_ has a normal plane *span*{*M*_1*μ*_^1^, *M*_2*μ*_^1^} at *μ* = 0.

(ii) there exists an (*l* − 1)-dimensional surface

(35) L12={μ:δ−1w212M2μ1μ+(δ−1M1μ1μ)ρ22/λ21+h.o.t.=0,     M1μ1μ>0}

(36) (resp.,  L21={μ:δ1−(ρ11)−1δln⁡⁡(−δ−1w112M1μ1μ)        −M2μ1μ+h.o.t.=0,  w112M1μ1μ<0})

such that system (1) has a unique homoclinic loop connecting *p*_1_ (resp., connecting *p*_2_) near Γ if and only if *μ* ∈ *L*_1_^2^ (resp., *μ* ∈ *L*_2_^1^).

**Proof**

The result (i) will be proved by putting *s*_1_ = *s*_2_ = 0 into (33).

If we assume *s*_1_ = 0 and *s*_2_ > 0 in (33), then

(37) $$\begin{array}{c} s_{2}^{\lambda_{2}^{1}/\rho_{2}^{1}}=\delta^{-1}M_{1\mu }^{1}\mu +\text{h}.\text{o}.\text{t}.,\\ {\left(w_{2}^{12}\right)}^{-1}s_{2}^{\rho_{2}^{2}/\rho_{2}^{1}}+\delta^{-1}M_{2\mu }^{1}\mu +\text{h}.\text{o}.\text{t}.=0, \end{array}$$

which means

(38) $$\begin{array}{c} {\left(w_{2}^{12}\right)}^{-1}s_{2}^{\rho_{2}^{2}/\lambda_{2}^{1}}+\delta^{-1}M_{2\mu }^{1}\mu +\text{h}.\text{o}.\text{t}.=0. \end{array}$$

It follows that there exists an (*l* − 1)-dimensional surface *L*_1_^2^ given by (35) such that (33) has a unique solution *s*_1_ = 0, *s*_2_ = *s*_2_(*μ*) > 0 as  *μ* ∈ *L*_1_^2^ and 0 < |*μ* | ≪1. This implies system (1) has a homoclinic loop connecting *p*_1_. The existence of *L*_2_^1^ can be obtained similarly.

This completes the proof.

**Remark 3**

There is no difficulty to see that *L*_1_^2^ has a normal vector *M*_2*μ*_^1^ at *μ* = 0 as *ρ*_2_^2^ > *λ*_2_^1^, while for *ρ*_2_^2^ < *λ*_2_^1^ (resp., *ρ*_2_^2^ > *λ*_2_^1^) it has a normal vector *M*_1*μ*_^1^ (resp., *M*_1*μ*_^1^ + *w*_2_^12^ *M*_2*μ*_^1^) at *μ* = 0.

**Theorem 4**

Assume the conditions (*H*_1_)–(*H*_3_) hold and *M*_*iμ*_^1^ ≠ 0, *i* = 1,2. Then for *λ* = 0, *μ* ∈ *L*_1_^2^, and 0 < |*μ* | ≪1, the periodic orbit and homoclinic loop with *p*_1_ of system (1) cannot coexist.

**Proof**

Theorem 2 shows that if *μ* ∈ *L*_1_^2^ and 0 < |*μ* | ≪1, then system (1) has a homoclinic loop with  *p*_1_. Setting *s*_1_ ≥ 0, *s*_2_^*λ*_2_^1^/*ρ*_2_^1^^ = (*w*_1_^12^)^−1^ *s*_1_ + *δ* ^−1^ *M*_1*μ*_^1^ *μ* + h.o.t.>0, and *μ* ∈ *L*_1_^2^, then (33) is reduced to

(39) V1(s1)≜[(w112)−1s1+δ−1M1μ1μ]ρ22/λ21+δ−1w212M2μ1μ+h.o.t.=w2121−(ρ11)−1δln⁡s1≜N1(s1).

Notice that *V*_1_(0) = *N*_1_(0) and

(40) $$\begin{array}{c} V_{1}^{\prime }\left(s_{1}\right)=\frac{\rho_{2}^{2}}{\lambda_{2}^{1}}{\left(w_{1}^{12}\right)}^{-1}{\left[{\left(w_{1}^{12}\right)}^{-1}s_{1}+\delta^{-1}M_{1\mu }^{1}\mu \right]}^{\rho_{2}^{2}/\lambda_{2}^{1}-1},\\ N_{1}^{\prime }\left(s_{1}\right)=\frac{w_{2}^{12}{\left(\rho_{1}^{1}\right)}^{-1}\delta }{{\left(1-{\left(\rho_{1}^{1}\right)}^{-1}\delta \ln s_{1}\right)}^{2}s_{1}}. \end{array}$$

If *w*_1_^12^ *w*_2_^12^ < 0, then *V*_1_′(*s*_1_)*N*_1_′(*s*_1_) < 0; it is obvious that *V*_1_(*s*_1_) = *N*_1_(*s*_1_) has no sufficiently small positive solutions.

While *ρ*_2_^2^ > *λ*_2_^1^, then |*V*_1_′(*s*_1_)|≪1 and |*N*_1_′(*s*_1_)|≫1 hold for 0 < *s*_1_ ≪ 1, which shows that *V*_1_(*s*_1_) = *N*_1_(*s*_1_) has no sufficiently small positive solution.

Next, we only consider the case *ρ*_2_^2^ ≤ *λ*_2_^1^ and *w*_1_^12^ *w*_2_^12^ > 0. As *μ* ∈ *L*_1_^2^, we have *M*_1*μ*_^1^ *μ* > 0, and then, for *w*_*i*_^12^ > 0, *i* = 1,2 we see that

(41) $$\begin{array}{c} V_{1}^{\prime }\left(s_{1}\right)\leq {\left(w_{1}^{12}\right)}^{-\rho_{2}^{2}/\lambda_{2}^{1}}s_{1}^{\rho_{2}^{2}/\lambda_{2}^{1}-1}<N_{1}^{\prime }\left(s_{1}\right)\;\text{for}\;\;0<s_{1}\ll 1. \end{array}$$

In fact, *ρ*_2_^2^ < *λ*_2_^1^ yields that lim⁡_*s*_1_→0^+^_ *s*_1_^*ρ*_2_^2^/*λ*_2_^1^−1^ = +*∞*, lim⁡_*s*_1_→0^+^_ *N*_1_′(*s*_1_) = +*∞*, and lim⁡_*s*_1_→0_ *s*_1_^*ρ*_2_^2^/*λ*_2_^1^−1^/*N*_1_′(*s*_1_) = 0, which shows *V*_1_(*s*_1_) = *N*_1_(*s*_1_) has no sufficiently small positive solutions. Obviously, the conclusion is correct as  *ρ*_2_^2^ = *λ*_2_^1^.

Similarly, for *ρ*_2_^2^ < *λ*_2_^1^, *w*_*i*_^12^ < 0, *i* = 1,2, there does not exist a small positive solution for *V*_1_(*s*_1_) = *N*_1_(*s*_1_).

The proof is then completed.

**Theorem 5**

Assume that the conditions (*H*_1_)–(*H*_3_) hold and *M*_*iμ*_^1^ ≠ 0, *i* = 1,2. Let *λ* = 0, *μ* ∈ *L*_2_^1^, and 0 < |*μ* | ≪1; then

- the periodic orbit and the homoclinic loop connecting *p*_2_ of system (1) cannot coexist as *ρ*_2_^2^ ≥ *λ*_2_^1^ or *w*_1_^12^ *w*_2_^12^ < 0;
- at least one periodic orbit and the homoclinic loop connecting *p*_2_ of system (1) coexist as *ρ*_2_^2^ < *λ*_2_^1^, *w*_1_^12^ > 0, and *w*_2_^12^ > 0;
- a unique periodic orbit and the homoclinic loop connecting *p*_2_ of system (1) coexist as *ρ*_2_^2^ < *λ*_2_^1^, *w*_1_^12^ < 0, and *w*_2_^12^ < 0.

**Proof**

By Theorem 2, the condition *μ* ∈ *L*_2_^1^  for 0 < |*μ* | ≪1 implies that system (1) has a homoclinic loop connecting *p*_2_.

(i) Let *s*_2_ = *e* ^−*ρ*_2_^1^*τ*_2_^ and eliminating  *s*_1_  in (33), we derive

(42) V2(s2)≜s2+δ−1w212M2μ1μ+h.o.t.=w2121−(ρ11)−1δln⁡(w112(s2λ21/ρ22−δ−1M1μ1μ))≜N2(s2).

Note that *V*_2_(0) = *N*_2_(0) as *μ* ∈ *L*_2_^1^. Moreover,

(43) V2′(s2)=1,N2′(s2)=w212(ρ11)−1δ[1−(ρ11)−1δln⁡(w112(s2λ21/ρ22−δ−1M1μ1μ))]2·(λ21/ρ22)s2(λ21−ρ22)/ρ22(s2λ21/ρ22−δ−1M1μ1μ).

For *ρ*_2_^2^ ≥ *λ*_2_^1^ and *N*_2_′(*s*_2_) ≪ 1 = *V*_2_′(*s*_2_), this means *V*_2_(*s*_2_) = *N*_2_(*s*_2_) has no sufficiently small positive solutions.

Now we turn to the case *w*_1_^12^ *w*_2_^12^ < 0, since we are interested in sufficiently small positive solutions of (33), it suffices to consider the sufficiently small positive solutions of *V*_2_(*s*_2_) = *N*_2_(*s*_2_) satisfying *w*_1_^12^(*s*_2_^*λ*_2_^1^/*ρ*_2_^2^^ − *δ* ^−1^ *M*_1*μ*_^1^ *μ*) > 0, which implies that *s*_2_^*λ*_2_^1^/*ρ*_2_^2^^ − *δ* ^−1^ *M*_1*μ*_^1^ *μ* < 0 (resp., *s*_2_^*λ*_2_^1^/*ρ*_2_^2^^ − *δ* ^−1^ *M*_1*μ*_^1^ *μ* > 0) for *w*_1_^12^ < 0 (resp., *w*_1_^12^ > 0). It is easy to see that *V*_2_(*s*_2_) = *N*_2_(*s*_2_) has no sufficiently small positive solutions as *w*_1_^12^ *w*_2_^12^ < 0.

(ii) For *ρ*_2_^2^ < *λ*_2_^1^, we have *V*_2_′(0) = 1 > 0 = *N*_2_′(0), which implies that there exists an $0<{\tilde{s}}_{2}\ll 1$ such that *V*_2_(*s*_2_) > *N*_2_(*s*_2_) for $0<s_{2}<{\tilde{s}}_{2}$.

Choosing ${\hat{s}}_{2}=\delta^{-1}w_{2}^{12}M_{2\mu }^{1}\mu >0$, then

(44) $$\begin{array}{c} V_{2}\left({\hat{s}}_{2}\right)=2\delta^{-1}w_{2}^{12}M_{2\mu }^{1}\mu +\text{h}.\text{o}.\text{t}.,\\ N_{2}\left({\hat{s}}_{2}\right)=\frac{w_{2}^{12}}{1-{\left(\rho_{1}^{1}\right)}^{-1}\delta \ln\left(w_{1}^{12}\left({\hat{s}}_{2}^{\lambda_{2}^{1}/\rho_{2}^{2}}-\delta^{-1}M_{1\mu }^{1}\mu \right)\right)}. \end{array}$$

In view of $\ln(w_{1}^{12}({\hat{s}}_{2}^{\lambda_{2}^{1}/\rho_{2}^{2}}-\delta^{-1}M_{1\mu }^{1}\mu ))>\ln(w_{1}^{12}{\hat{s}}_{2}^{\lambda_{2}^{1}/\rho_{2}^{2}})=\ln(w_{1}^{12}{\left(\delta^{-1}w_{2}^{12}M_{2\mu }^{1}\mu \right)}^{\lambda_{2}^{1}/\rho_{2}^{2}})$ for *w*_1_^12^ > 0, so

(45) N2(s^2)>w2121−(ρ11)−1δln⁡(w112(w112(δ−1w212M2μ1μ)λ21/ρ22))≫2w112(δ−1w212M2μ1μ)=V2(s^2)

when *w*_2_^12^ > 0. As a result, *N*_2_(*s*_2_) = *V*_2_(*s*_2_) has at least one solution ${\overset{-}{s}}_{2}$ satisfying $0<{\tilde{s}}_{2}<{\overset{-}{s}}_{2}<{\hat{s}}_{2}\ll 1$.

(iii) *s*_2_ must fulfill 0 < *s*_2_ < (*δ* ^−1^ *M*_1*μ*_^1^ *μ*)^*ρ*_2_^2^/*λ*_2_^1^^ as *w*_1_^12^ < 0; with similar arguments in proof of (ii), we can prove that there exists a 0 < *s*_2_* ≪ 1 such that *V*_2_(*s*_2_*) = *N*_2_(*s*_2_*) for 0 < *s*_2_* < (*δ* ^−1^ *M*_1*μ*_^1^ *μ*)^*ρ*_2_^2^/*λ*_2_^1^^ ≪ 1. It is easy to compute that *N*_2_′′(*s*_2_) > 0 for *w*_2_^12^ < 0, 0 < *s*_2_ < (*δ* ^−1^ *M*_1*μ*_^1^ *μ*)^*ρ*_2_^2^/*λ*_2_^1^^, and *μ* ∈ *L*_2_^1^. Combining with the fact *V*_2_(0) = *N*_2_(0), *N*_2_′(*s*_2_) > 0, and *V*_2_′(*s*_2_) = 1, we immediately know that *s*_2_* is unique.

This completes the proof.

Now, we turn to discussing the bifurcations of the heteroclinic loop for *λ* > 0, when *p*_1_ undergoes a transcritical bifurcation. From Figure 1, we know that when *λ* > 0, after the creation of the equilibria *p*_1_^0^ and *p*_1_^1^, there always exists a straight segment orbit heteroclinic to *p*_1_^1^ and *p*_1_^0^, its length is *λ*_*p*_, and we denote this heteroclinic orbit by Γ*. Moreover, *x*_0_^1^ = *λ*_*p*_ is a critical position.

Firstly, we take into account the case *x*_0_^1^ ≥ *λ*_*p*_. In this case, (31) becomes

(46) (w112)−1δs1−δs2λ21/ρ21+M1λ1λ+M1μ1μ+h.o.t.=0,(w212)−1δs2ρ22/ρ21−δλp[δ−(δ−λp)s1λp/ρ11]−1 +M2λ1λ+M2μ1μ +w231(w233)−1s1λ11/ρ11[w113(w112)−1δs1+(w133)−1δs2λ22/λ21          −M1λ3λ−M1μ3μ]+h.o.t.=0.

Let *s* = *s*_1_^*λ*_*p*_/*ρ*_1_^1^^ (*s* = 0 means *s*_1_ = 0 and vice versa); by virtue of Taylor's development for *δλ*_*p*_/(*δ* − (*δ* − *λ*_*p*_)*s*_1_^*λ*_*p*_/*ρ*_1_^1^^), we have

(47) (w112)−1sρ11/λp−s2λ21/ρ21+δ−1M1λ1λ+δ−1M1μ1μ+h.o.t.=0,(w212)−1δs2ρ22/ρ21−λp−λp(δ−λp)δs+M2λ1λ  +M2μ1μ+h.o.t.=0.

With similar arguments to *λ* = 0, we may easily obtain the following results.

**Theorem 6**

Suppose the conditions (*H*_1_)–(*H*_3_) hold, 0 < *λ* ≪ 1; then

(i) if rank⁡(*M*_1*μ*_^1^, *M*_2*μ*_^1^) = 2, there exists an  (*l* − 2)-dimensional surface

(48) L12λ={μ(λ):M1μ1μ+M1λ1λ+h.o.t.    =M2μ1μ+M2λ1λ−λ+h.o.t.=0}

such that system (1) has a unique heteroclinic loop if and only if *μ* ∈ *L*_12_^*λ*^ and 0 < |*μ* | ≪1;

(ii) there exists an (*l* − 1)-dimensional surface

(49) L1λ2={μ(λ):W12(λ,μ)=(w212)−1[δ−1(M1μ1μ+M1λ1λ)]β2            +δ−1(M2μ1μ+M2λ1λ)            −δ−1λp+h.o.t.=0,       M1μ1μ+M1λ1λ>0}(resp., L2λ1={μ(λ):W21(λ,μ)       =δλp+λp(δ−λp)        ×[−δ−1w112(M1λ1λ+M1μ1μ)]λp/ρ11        −δM2λ1λ        −δM2μ1μ+h.o.t.=0,       w112(M1λ1λ+M1μ1μ)<0})

such that system (1) has one homoclinic loop connecting *p*_1_^1^ (resp., connecting *p*_2_) if and only if *μ* ∈ *L*_1*λ*_^2^ and 0 < |*μ* | ≪1.

**Theorem 7**

Suppose hypotheses (*H*_1_)–(*H*_3_) hold, *M*_*iμ*_^1^ ≠ 0, *i* = 1,2, 0 < *λ*, |*μ* | ≪1, and *w*_1_^12^ *w*_2_^12^ < 0. Then, except the homoclinic loop connecting *p*_1_^1^ (resp., *p*_2_), system (1) has no periodic orbits as *μ* ∈ *L*_1*λ*_^2^ (resp., *μ* ∈ *L*_2*λ*_^1^).

**Remark 8**

It is easy to see that homoclinic loop connecting *p*_1_^0^ and heteroclinic loop joining *p*_1_^0^, *p*_2_ cannot be bifurcated from Γ, which is exactly determined by the generic condition (*H*_1_).

Finally, we consider the case −*β* ≤ *x*_0_^1^ < *λ*_*p*_. Due to Figure 1 and (25), it follows from (31) that

(50) $$\begin{array}{c} s_{2}={\left[\delta^{-1}\left(M_{1\lambda }^{1}\lambda +M_{1\mu }^{1}\mu \right)\right]}^{\rho_{2}^{1}/\lambda_{2}^{1}}+\text{h}.\text{o}.\text{t}.,\\ x_{0}^{1}={\left(w_{2}^{12}\right)}^{-1}\delta s_{2}^{\rho_{2}^{2}/\rho_{2}^{1}}+M_{2\lambda }^{1}\lambda +M_{2\mu }^{1}\mu +\text{h}.\text{o}.\text{t}. \end{array}$$

**Theorem 9**

Assume the conditions (*H*_1_)–(*H*_3_) are true, rank⁡(*M*_1*λ*_^1^, *M*_1*μ*_^1^) > 0 and rank⁡(*M*_2*λ*_^1^, *M*_2*μ*_^1^) > 0. Then,

(i) there exists a surface
  (51) Σ1(μ,λ)={μ(λ):[δ−1(M1λ1λ+M1μ1μ)]ρ21/λ21+h.o.t.=0,   −β≤M2λ1λ+M2μ1μ+h.o.t.<λp,   0<|μ|, λ≪1},
  such that system (1) has two orbits heteroclinic to *p*_1_^1^, *p*_2_, *p*_1_^0^ as *μ* ∈ Σ_1_(*μ*, *λ*);
(ii) there exists a region in the (*λ*, *μ*) space
  (52) Δ={(λ,μ):−β≤(w212)−1δ(λ21−ρ22)/λ21      ×(M1λ1λ+M1μ1μ)ρ22/λ21      +M2λ1λ+M2μ1μ      +h.o.t.<λp,    M1λ1λ+M1μ1μ>0,    0<|μ|,  λ≪1},
  such that system (1) has a heteroclinic orbit connecting *p*_1_^1^ and *p*_1_^0^ for (*λ*, *μ*) ∈ Δ.

**Proof**

(i) If *s*_2_ = 0 in (50), then

(53) $$\begin{array}{c} 0={\left[\delta^{-1}\left(M_{1\lambda }^{1}\lambda +M_{1\mu }^{1}\mu \right)\right]}^{\rho_{2}^{1}/\lambda_{2}^{1}}+\text{h}.\text{o}.\text{t}.,\\ x_{0}^{1}=M_{2\lambda }^{1}\lambda +M_{2\mu }^{1}\mu +\text{h}.\text{o}.\text{t}. \end{array}$$

which shows that there exists a surface Σ_1_(*μ*, *λ*) such that (50) has a solution *s*_2_ = 0 and −*β* ≤ *x*_0_^1^ < *λ*_*p*_ for *μ* ∈ Σ_1_(*μ*, *λ*), then system (1) has two heteroclinic orbits, one is heteroclinic to  *p*_1_^1^  and  *p*_2_ and the other is heteroclinic to *p*_2_ and  *p*_1_^0^.

(ii) If *s*_2_ > 0 in (50), one attains *M*_1*λ*_^1^ *λ* + *M*_1*μ*_^1^ *μ* > 0. Eliminating *s*_2_ in (50), we achieve

(54) x01=(w212)−1δ(λ21−ρ22)/λ21(M1λ1λ+M1μ1μ)ρ22/λ21+M2λ1λ+M2μ1μ+h.o.t.,

which shows that there exists a region Δ such that when (*λ*, *μ*) ∈ Δ, system (1) has one heteroclinic orbit heteroclinic to *p*_1_^1^ and *p*_1_^0^.

**Remark 10**

All the heteroclinic orbits joining *p*_1_^0^ will go into *p*_1_^0^ in different ways according to different fields of *x*_0_^1^; see Figure 2.

## Figures and Tables

**Figure 2:**